# Successful Treatment of Cutaneous Squamous Cell Cancer with Cemiplimab—A Report of Two Cases Demonstrating the Management of Pseudoprogression and Adverse Events

**DOI:** 10.3390/jcm13144236

**Published:** 2024-07-19

**Authors:** Paulina Żukowska, Katarzyna Ciepiela, Aleksandra Kudrymska, Kajetan Kiełbowski, Rafał Becht

**Affiliations:** 1 Department of Clinical Oncology, Chemotherapy and Cancer Immunotherapy, Pomeranian Medical University, 71-252 Szczecin, Poland; paulina.a.zukowska@gmail.com (P.Ż.); katarzyna.ciepiela@usk1.szczecin.pl (K.C.); kajetan.kielbowski@onet.pl (K.K.); 2 Department of Pathology, Pomeranian Medical University, 71-252 Szczecin, Poland; olaqdr@wp.pl

**Keywords:** squamous cell cancer, immunotherapy, immune checkpoint inhibitors, programmed cell death 1 receptor

## Abstract

**Background**: Cutaneous squamous cell carcinoma is a common malignancy, which frequently develops in the areas exposed to the sun. Patients with locally advanced disease in the head and neck region are frequently disqualified from surgical resection and require systemic treatment. **Methods**: In this report, we present the clinicopathological features and treatment of two patients who received cemiplimab, a monoclonal antibody targeting programmed cell death receptor 1 (PD-1). **Results**: An 80-year-old female and 82-year-old male patient were admitted to the hospital for the treatment of large tumors diagnosed as squamous cell carcinomas. In both patients, surgical treatment was not recommended due to the large dimensions of the tumors. These patients qualified for systemic treatment with cemiplimab. In the first patient, immunotherapy was interrupted due to adverse events. Nevertheless, a continuous regression of the tumor was observed despite treatment cessation. The second patient experienced a pseudoprogression, which is an increase in the tumor size caused by infiltration of immune cells. The treatment significantly reduced tumor size in both patients, which highly improved their quality of life. **Conclusions**: Cemiplimab offers clinical benefits in patients with cutaneous squamous cell carcinoma who are ineligible for surgical treatment. Systemic treatment can significantly improve the quality of life and reduce tumor diameters.

## 1. Introduction

Non-melanoma skin cancers are the most common malignant tumors in humans [1,2]. The second most frequently diagnosed skin cancer, after basal cell carcinoma, is squamous cell carcinoma (SCC). It originates from spindle cells, making up the stratum spinosum of the epidermis. The incidence of the disease increases with age, with the highest risk in individuals over 60 years old [3]. The majority of these cancers are located on the skin exposed to solar radiation, such as the face, auricles, scalp, as well as upper and lower limbs. Squamous cell carcinoma is most often characterized by local growth, with rare distant metastases. If untreated, it leads to the destruction of skin, soft tissues, cartilages, and bones. However, due to unfavorable location or advanced clinical stage, surgical treatment may not be possible. Immunotherapy with cemiplimab, an anti-programmed cell death protein 1 (PD-1) antibody, has been approved for patients ineligible for radical surgery or radiotherapy. The aim of this paper is to present two patients with cutaneous SCC that were treated with cemiplimab. We discuss the management of adverse events (AEs) and pseudoprogression. Moreover, we aim to demonstrate the benefits of systemic immunotherapy in patients who are not candidates for surgical treatment to physicians of other specialties.

## 2. Case Report

### 2.1. Case 1

**Figure 1:**
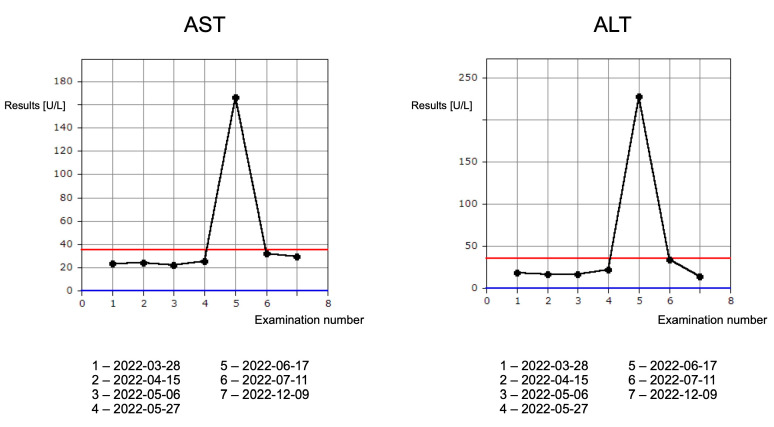
Graphs demonstrating fluctuations of AST and ALT laboratory parameters. Results noted in U/L (normal range 0–35 U/L). Blue and red lines indicate normal range. The longer interval before the last measurement results from a distant place of residence of the patient. Some of the results were performed in her hometown.

In October 2021, an 80-year-old female patient, previously treated only for hypertension, underwent a surgical biopsy of a skin lesion on her lower lip. Based on histopathological examination, keratinizing squamous cell carcinoma G2 was diagnosed. Local recurrence with rapid growth was observed in December 2021. A pathological lesion of the lower lip measuring 26 × 36 × 24 mm was identified during a contrast-enhanced computed tomography examination of the craniofacial region performed in January 2022. The lesion (adjacent to the base of the tongue) underwent strong, heterogeneous contrast enhancement, and infiltrated the skin, subcutaneous tissue, mimic muscles, ipsilateral gum as well as the mucosa of the bottom of the mouth and the cheek. Ultrasound examination of cervical lymph nodes did not confirm the presence of pathological infiltrations. Abdominal ultrasonography and computed tomography (CT) of the chest did not reveal any suspicious lesions. In March 2022, the patient had an oncology consultation, where the diagnosis of keratinizing squamous cell carcinoma G2 at the cT3N0M0 stage was confirmed. Clinically, the lesion measured approximately 4 cm. Neither radical surgical treatment nor radical radiotherapy was possible due to the significant risk of disability and too of a wide field requiring irradiation, respectively. Consequently, in the absence of contraindications, the patient was qualified for a cyclic treatment with 350 mg i.v. cemiplimab, an anti-PD-1 antibody, at three-week intervals. The first administration of the drug was well tolerated, without clinically noticeable consequences. However, a grade 1 hyperthyroidism (Common Terminology Criteria for Adverse Events, CTCAE [4]) was diagnosed prior to the second drug administration. Based on the endocrinology consultation, treatment with thiamazole was introduced and, due to the lack of contraindications, immunotherapy was continued. At the same time, a gradual reduction of the skin lesion was observed, which decreased in its largest dimension to 20 mm after the third administration of the drug. In laboratory tests, normalization of peripheral thyroid hormone levels was observed at that time, with persistently low TSH levels. Before the fourth drug administration, the patient complained of pain in the right subcostal region and abdominal bloating. In laboratory tests, CTCAE grade 3 increased transaminase activity was observed (Figure 1), with a normal bilirubin level, albumin level, and INR.

**Figure 2:**
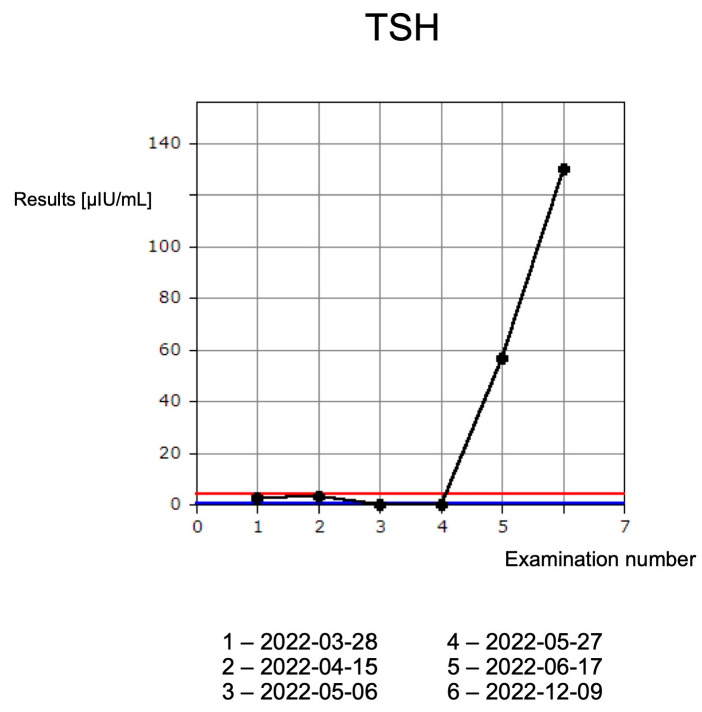
A graph demonstrating fluctuations of TSH laboratory parameters. Results noted in µIU/mL (normal range 0.27–4.2 µIU/mL). Blue and red lines indicate normal range. The longer interval before the last measurement results from a distant place of residence of the patient. Some of the results were performed in her hometown.

Contrary to the time before the second administration of the drug, CTCAE grade 1 hypothyroidism was diagnosed (Figure 2).

**Figure 3:**
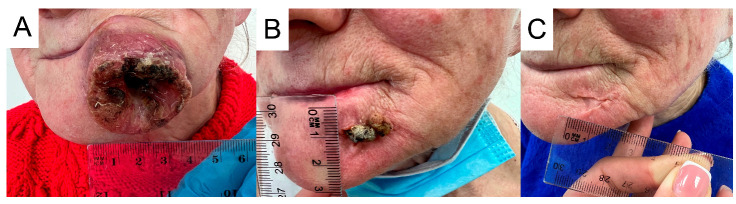
Squamous cell carcinoma of patient 1. (**A**) 28 March 2022. (**B**) 17 June 2022. (**C**) 9 December 2022.

In the absence of other premises, the liver damage was considered to be immune-related. Therefore, the fourth administration of cemiplimab and treatment with thiamazole were discontinued. In the treatment of complications, oral prednisone was introduced at the initial dose of 1 mg/kg bw/day (with subsequent tapering); L-thyroxine and hepatoprotective drugs were also introduced. The treatment turned out to be fully effective and it led to the normalization of all laboratory parameters (except TSH) and the resolution of symptoms. The TSH level was above normal. What is extremely important, despite immunotherapy cessation, is that a continuous regression of the skin lesion was observed in subsequent follow-up observations into the form of a small triangular scar (Figure 3). Follow-up in December revealed the patient’s non-compliance to levothyroxine treatment, but without significant clinical implications. Currently, the patient is under the supervision of the Endocrinology Clinic and the family physician; no progression of facial cancer is being observed. Due to the significant regression of facial lesions, advanced age and the distant place of residence of the patient, she is being consulted every 6 months by the oncologist.

### 2.2. Case 2

**Figure 4:**
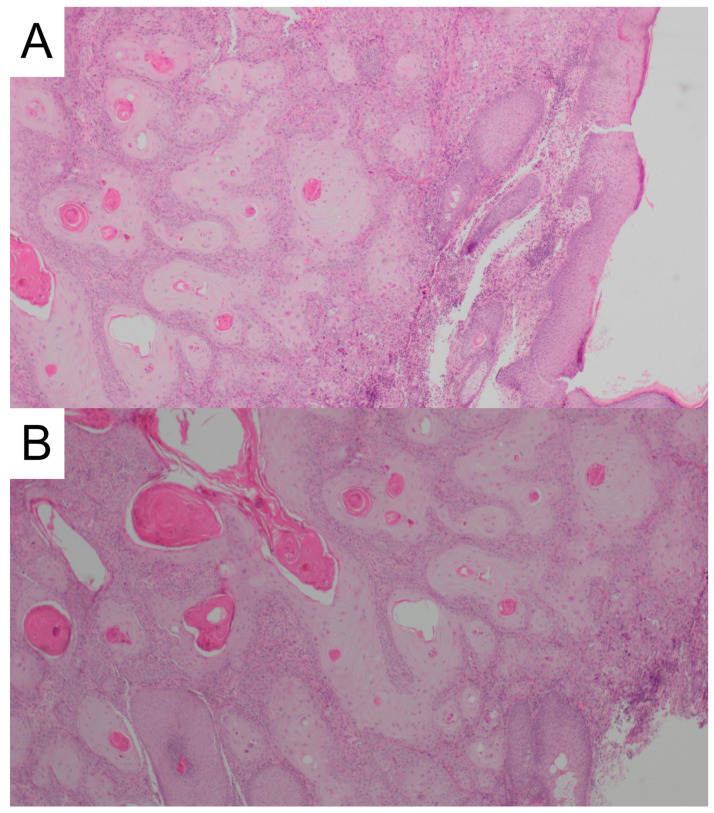
(**A,B**) Histopathology of the cutaneous squamous cell carcinoma of patient 2, H&E staining (40×).

**Figure 5:**
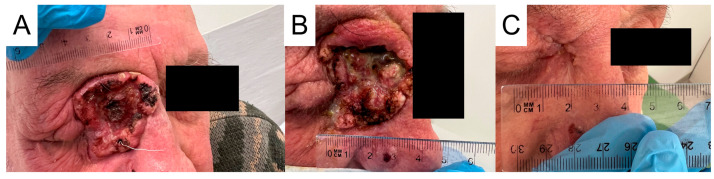
Squamous cell carcinoma of patient 2. (**A**) 13 March 2023. (**B**) 5 May 2023. (**C**) 18 August 2023.

In February 2022, an 82-year-old male patient observed an itching nodule in the inner canthus of the right eye. The patient had a medical history of arterial hypertension, type 2 diabetes, and respiratory failure requiring hospitalization in the intensive care unit due to SARS CoV-2 pneumonia in January 2021. The diagnosis process was initiated when the primary lesion was quite large and further lesions appeared in the fronto-parietal area on the right side and the right nasal wing. Histopathological examination of the samples collected from the above-mentioned lesions confirmed the diagnosis of keratinizing squamous cell carcinoma G2 in the eye canthus (Figure 4) and nodular basal cell carcinoma at the other sites. In February 2023, CT of the craniofacial region showed contrast-enhancing nodular masses in the skin of the nasal wing, medial canthus of the right eye reaching the lacrimal canal and the right frontal region. Furthermore, it confirmed a pathologically enlarged lymph node in the anterior pole of the right parotid gland. Abdominal ultrasonography and chest CT did not show any lesions that would raise oncological concerns. The multidisciplinary oncology team decided that surgical treatment and radiotherapy would be associated with significant mutilation of the patient and loss of the eyeball. Consequently, the patient was qualified for systemic treatment with cemiplimab at a standard dose of 350 mg i.v. at three-week intervals. The treatment was started in March 2023, approximately a year after the patient noticed the first skin lesions. At that time, the largest lesion was the crater-shaped tumor, approximately 4 cm in diameter. Heavy swelling caused vision impairment and difficulties in the opening of eyelids. Initially, the lesion did not decrease. On the contrary, by the seventh week of treatment, it appeared to be larger. Moreover, an enlarged parotid lymph node brought oncological concerns. A control CT examination of the craniofacial region revealed the enlargement of the infiltration in the area of the medial canthus of the right eye to 4.5 cm in the cranio-caudal (CC) dimension. In addition, a significant increase, as much as threefold, in size of the lymph node in the right parotid gland was found. At the same time, the tolerance of immunotherapy was very good, and no side effects were observed. Enlargement of the lesion was considered neoplastic pseudoprogression and continuation of treatment was recommended. As expected, three weeks later, the lesion underwent a clear regression—it became shallower, the boundaries of the lesion were smoothed, and its diameter significantly decreased. The size of the parotid lymph node was also reduced. At the moment, the treatment is continued without complications, and the skin lesion undergoes further clear regression. At the same time, the swelling of the eyelid decreased and the palpebral fissure widened (Figure 5). CT scan from September 2023 confirmed regression of the SCC infiltration. The length of the lesion (craniocaudal) was 4.2 cm (compared to 4.5 cm in June) and the depth was 1.1 cm (compared to 1.6 cm in May). Moreover, the examination confirmed reduced dimensions of the lymph nodes located in the parotid gland (2.4 × 1.5 cm vs 2.8 × 2 cm and 6 mm vs 11 mm). The patient is still being treated with cemiplimab and a CT is performed every 12 weeks. The patient has received a total of 22 cycles and the most recent CT performed on 14 June 2024 confirmed a partial response (PR).

## 3. Discussion

**Figure 6:**
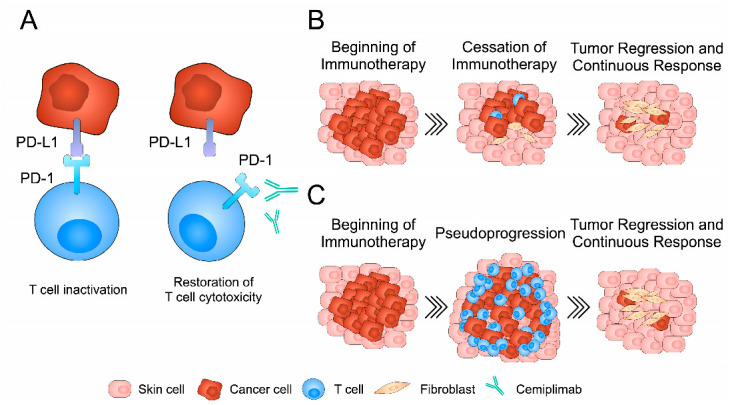
A schematic illustration of the mechanism of action of cemiplimab and responses observed in described patients. (**A**) Cemiplimab binds to PD-1 receptor present on the surface of the T cells and prevents PD-1/PD-L binding. (**B**) Tumor regression in patient 1 was observed after discontinuation of cemiplimab treatment. (**C**) Pseudoprogression could be observed in patient 2 during the immunotherapy, which was followed by the shrinkage of the lesion.

Cemiplimab is a fully human monoclonal antibody (IgG4 immunoglobulin) directed against the PD-1. PD-1 is a membrane protein receptor and is expressed by T and B cells, as well as dendritic cells and macrophages [5,6,7]. PD-1 activation occurs after binding to one of the ligands: PD-L1 and PD-L2. Under physiological conditions, PD-L/PD-1 binding inhibits the activity of the immune system, thus preventing autoimmunity [8]. PD-L1 and PD-L2 present in cancer cells inhibit T cell functions such as proliferation, cytokine secretion, and cytotoxicity, leading to the uncontrolled proliferation of cancer cells. Cemiplimab, by blocking the binding of PD-1 to the ligands PD-L1 and PD-L2, enhances the response of T lymphocytes and restores the anti-cancer activity of the immune system (Figure 6).

Cemiplimab was approved for the treatment of patients with cutaneous SCC (CSCC) in September 2018 in the United States, and in July 2019 in Europe. Moreover, cemiplimab is included as one of the options for systemic treatment in the National Comprehensive Cancer Network (NCCN) guidelines for squamous cell skin cancer. The second immunotherapeutic recommended for the treatment of advanced CSCC is pembrolizumab [9,10]. Recently, nivolumab demonstrated promising efficacy in patients with metastatic or locally advanced CSCC as well [11].

PD-1 inhibitors should be reserved for patients in which a safe curative surgical approach and/or radiation are not possible [12]. The FDA approval of cemiblimab was based on the phase I (NCT02383212) and phase II (EMPOWER-CSCC-1; NCT02760498) studies, which showed a significant anti-cancer activity of this immunotherapeutic. Treatment response rates were 50% in the group of 26 patients in the phase I study (locally advanced and metastatic disease) and 47% in the group of 59 patients in the phase II study (metastatic disease). In the group of patients responding to treatment, responses were long-lasting and exceeded 6 months in 57% of patients. Adverse events occurred in 15% of patients, and only 7% of patients discontinued treatment for this reason [13]. In the subsequent analysis of the 78 patients with locally advanced disease from the phase II study, the objective response was observed in 44% of patients. In this group, grade 3–4 treatment-emergent AEs were observed in 44% of patients. Grade 1–2 AEs developed in 99% of patients and the most common included fatigue (41%), diarrhea (27%), and pruritus (27%). Hypothyroidism occurred in 10% of patients [14]. Overall, a total of 193 patients divided into 3 groups were analyzed in this study. The objective response rate (ORR) was 47.2% and the median progression-free survival (PFS) was 22.1 months. The median overall survival (OS) was not reached [15]. Drugs targeting immune checkpoints have specific, immuno-related adverse events (irAE) resulting from the aggravation of autoimmune reactions leading to autoimmune complications such as skin lesions, pneumonia, colitis, nephritis, endocrine disorders, hepatitis, uveitis, arthritis, myositis, pancreatitis, severe skin reactions, Guillain–Barré syndrome, myasthenic syndrome, and hemolytic anemia [16]. The treatment of irAE has been strictly defined. Patient education, interdisciplinary cooperation, treatment in accordance with therapeutic algorithms, and the earliest possible introduction of corticosteroids allow for proper management of side effects without the need to discontinue therapy. Nevertheless, in cases of steroid-associated AEs or those in need of chronic corticosteroid therapy, other immunomodulatory agents may be required. Recently, Huang and colleagues reported a patient treated with cemiplimab who developed an ocular irAE. Despite corticosteroids and classic immunosuppressants, the patient was also treated with adalimumab, a tumor necrosis factor-alpha (TNF-α) inhibitor [17]. The efficacy of cemiplimab has also been confirmed in real-life studies. Baggi and collaborators analyzed 131 patients with advanced CSCC, who achieved an ORR of 58%. Interestingly, the response was better if the tumor was located in the head and neck region. Conversely, previous chemo- or radiotherapy, as well as a more advanced performance status (≥1), were associated with a poorer response [18]. Recently, McLean and colleagues reported an experience of using immune checkpoint inhibitors in patients with advanced CSCC. The study analyzed 286 patients, among whom 270 (94%) received cemiplimab. In total, 60% of included patients achieved the ORR, while the 12-month OS and PFS were 78% and 65%, respectively [19].

The first patient developed endocrine and hepatotoxic irAEs. An initial diagnosis of CTCAE grade 1 hyperthyroidism with progression to hypothyroidism is characteristic of autoimmune thyroiditis. After the introduction of thyrostatic therapy, levels of the free thyroid hormones decreased, and the patient could continue immunotherapy. After the third administration of cemiplimab, the patient developed hepatotoxicity, i.e., CTCAE grade 3 increase in transaminase activity, which was accompanied by pain in the right subcostal region. After the exclusion of other causes of hepatic cell damage, cemiplimab therapy was permanently discontinued, according to the algorithm of irAE management. Despite treatment discontinuation, the skin lesion continued to regress and 12 months after the last administration of cemiplimab, no progression of the neoplastic disease was observed. Due to the applied therapy, a significant improvement in quality of life was obtained. Previously, the patient avoided leaving the house due to the large lesion on the lower lip. The treatment enabled the patient to engage in a social life. Objective assessment of treatment response is possible with the use of imaging tests. RECIST criteria (version 1.1) are usually used in daily practice and clinical trials [20]. RECIST criteria were developed in 2000 when cytotoxic drugs were the backbone of oncological treatment. The mechanism of action of immunotherapy is different than standard cytotoxic therapy; therefore, the response to treatment may be different. More delayed and longer responses are observed, even after treatment discontinuation [21]. Tumor shrinkage or continuous response after the discontinuation of PD-1/PD-L1 inhibitors has been previously reported [22].

Pseudoprogression is a typical phenomenon for immunotherapy, which consists of the initial increase in the size of neoplastic lesions and/or the appearance of new foci after treatment initiation, and then their reduction during further therapy. This is due to the infiltration of immune cells (mainly T lymphocytes) within the neoplastic lesions, which leads to a temporary enlargement of the tumor mass. In the case of pseudoprogression, the increase in size is not due to the increase in the number of tumor cells, which distinguishes this phenomenon from true progression. For this reason, radiological control and assessment of treatment effectiveness should be performed not earlier than after three months of immunotherapy [23]. In the second case report, the phenomenon of pseudoprogression preceded the response to cemiplimab therapy and made it difficult to estimate the effectiveness of treatment at an early stage. Despite the enlargement of the neoplastic lesion and the increase in parotid lymph nodes on the right side in the seventh week of therapy, it was decided to continue the treatment. At subsequent visits, the reduction of the skin lesion and the parotid lymph node was observed. The patient continues cemiplimab therapy with good tolerance, with no signs of disease progression. Recently, Oliveira et al. described an interesting treatment response of a patient with metastatic CSCC treated with nivolumab. After 3 months of treatment, PET-CT examination revealed a reduction in the size of visceral lesions but confirmed new hypermetabolic areas in the spine. The patient continued the treatment and follow-up after 5 months revealed a metabolic response in the spinal lesions [24]. The occurrence of pseudoprogression is a known potential response to immunotherapy. However, it seems that it is a rare development in patients treated with cemiplimab. In a recent retrospective study published by Cañueto et al., the authors analyzed 83 patients with CSCC who were treated with systemic cemiplimab. Only three patients developed pseudoprogression [25]. As a result, evaluating a response during the immunotherapeutic treatment may be challenging due to atypical responses.

Interestingly, recent studies started to investigate the appropriate duration of cemiplimab treatment, together with its use in neoadjuvant settings, as well as in organ transplant recipients who are treated with immunosuppressive agents. Firstly, in an analysis by Khelef et al., the authors investigated the treatment courses with cemiplimab of 46 patients with advanced or metastatic CSCC who achieved a complete response (CR), followed by a cessation of immunotherapy. The authors evaluated that after achieving CR, the median time of treatment was approximately 3 months. Importantly, CR remained in all patients at the follow-up 17 months later [26]. In another recent study, Bailly-Caille and colleagues describe clinical courses of 14 patients treated with cemiplimab due to CSCC. Five patients were also treated with concomitant radiotherapy. The following response profile was noted in this cohort at the end of cemiplimab treatment: CR—9/14, PR—3/14, SD—1/14, and PD—1/14. After 24 months from treatment cessation, these ratios were as follows: CR—6/10, PR—2/10, SD—1/10, and PD—1/10. Two patients from the group died during the study period [27].

Secondly, a recent phase II trial demonstrated that cemiplimab may find use in the neoadjuvant setting. Among 20 patients with newly diagnosed or recurrent CSCC in the head and neck region (stage III-IV), 15 (75%) achieved a pathologic complete response or major pathologic response [28]. In another trial, seventy-nine patients with stage II-IV CSCC were included. Forty patients achieved a complete pathological response, and then a major pathological response. Importantly, at 24 months, an 85% event-free survival was achieved [29]. In addition, in a recent report by Geidel et al., the authors demonstrate that after 4 weeks of cemiplimab treatment at the standard dosage (350 mg every three weeks), a large 10 cm CSCC lesion reduced its mass by 90%. Subsequently, the lesion was resected (pathological complete response) and the patient was relapse-free in the follow-up at 20 months [30]. Moreover, cemiplimab has been investigated in patients with advanced CSCC after organ transplantation. Commonly used immunosuppressive agents that prevent graft rejection are considered to inhibit tumor surveillance, which is predisposed to carcinogenesis. Interestingly, according to a study by Hanna et al., out of 11 patients treated with cemiplimab and immunosuppressive therapy, 5 (46%) achieved a response, while the median PFS and OS were 22.5 each [31].

Importantly, cemiplimab has been approved for the treatment of patients with metastatic or locally advanced basal cell cancer (BCC), who previously failed treatment with hedgehog pathway inhibitors (HHI). Recently, Lewis et al. published a study that summarized the treatment of 54 patients with metastatic BCC previously treated with HHI. Two and ten patients achieved CR and PR, respectively. These results indicated that 22% of patients achieved ORR. Interestingly, 3 months was the calculated median time of response [32].

In rare cases, a single skin tumor can possess features of both SCC and BCC. Studies demonstrated the potential role of cemiplimab in the treatment strategies of such patients [33,34]. However, knowledge about the use of cemiplimab in these patients is limited [35]. Cemiplimab could be used sequentially with the hedgehog pathway inhibitors that are registered for the treatment of BCC. Proietti et al. describe a patient diagnosed with SCC and treated with cemiplimab, who achieved a partial metabolic response after twelve weeks of treatment. Subsequently, a revision of the primary tumor sample revealed BCC features, which led to the discontinuation of cemiplimab and the introduction of sonidegib. Consequently, the patient achieved a complete metabolic response [34]. Furthermore, concomitant use of cemiplimab and sonidegib showed promising efficacy in a patient with synchronous cutaneous SCC and BCC [36].

Importantly, some patients do not respond to immunotherapy, while others eventually develop acquired resistance. Various factors may contribute to the sensitivity to immunotherapy, but the composition of the tumor microenvironment (TME) is considered to play a significant role. Malignant cells interact with various cells surrounding the tumor tissue, including T cells, macrophages, or fibroblasts, among others. The expression of PD-L1, the presence of the M2 immunosuppressive macrophages, as well as different profiles of T cells, could affect treatment response [37]. Zou et al. analyzed the TME of CSCC tissues and found 11 subtypes of lymphocytes, 8 subtypes of dendritic cells, and 6 subpopulations of fibroblasts [38]. Intriguingly, recurrent CSCC tumors demonstrate different TME populations than primary tumors. For instance, tissues from relapsed patients show reduced presence of T cells. Furthermore, an elevated exhaustion score was observed in CD8+ cytotoxic T cells derived from recurred tumors compared to the primary tissues [39]. Future studies are required to investigate the impact of TME and its modification on response to cemiplimab/pembrolizumab treatment in patients with advanced CSCCs.

## 4. Conclusions

To conclude, cemiplimab showed significant efficacy and good tolerance in clinical trials and real-life studies. As a result, it is a recommended treatment option in patients with disseminated and locally advanced CSCC, when radical local treatment is impossible. In the described patients, treatment with cemiplimab significantly shrank the tumors and improved the quality of life of both patients. Appropriate management of side effects allows for the continuation of immunotherapy in the majority of cases reported in the literature. The knowledge of the phenomenon of pseudoprogression in the initial stage of immunotherapy treatment prevents too hasty therapy termination. We strongly believe that the potential cessation of treatment with cemiplimab is an important area of research that should be further investigated in the following years. As demonstrated in the first patient, the administration of only a few cycles resulted in a rapid and long-term response. More recent publications also start demonstrating the long-term responses after cessation of cemiplimab treatment. As with other malignancies and fields of medicine, searching for markers of response is a significant research area that would allow for personalized treatment selection and presumably improved outcomes. The presence of worse performance status (ECOG 2-3) and distant metastasis was associated with worse response in CSCC patients treated with cemiplimab [25]. Age has been suggested to be another important parameter that could correlate with response. As demonstrated by Ksienski and colleagues, the ORR was similar in three age groups. However, in the cohort including the oldest patients (≥85 years), a much higher fraction achieved progressive disease as the best overall response. Moreover, neutrophil-to-lymphocyte and platelet-to-lymphocyte ratios were associated with differences in survival [40]. Future studies are greatly needed to identify novel markers of response.

## Data Availability

The original contributions presented in the study are included in the article, further inquiries can be directed to the corresponding author.
